# Novel B19-Like Parvovirus in the Brain of a Harbor Seal

**DOI:** 10.1371/journal.pone.0079259

**Published:** 2013-11-05

**Authors:** Rogier Bodewes, Ana Rubio García, Lidewij C. M. Wiersma, Sarah Getu, Martijn Beukers, Claudia M. E. Schapendonk, Peter R. W. A. van Run, Marco W. G. van de Bildt, Marjolein J. Poen, Nynke Osinga, Guillermo J. Sánchez Contreras, Thijs Kuiken, Saskia L. Smits, Albert D. M. E. Osterhaus

**Affiliations:** 1 Department of Viroscience, Erasmus Medical Centre, Rotterdam, the Netherlands; 2 Seal Rehabilitation and Research Centre, Pieterburen, the Netherlands; 3 Division of Diagnostic Imaging, Faculty of Veterinary Medicine, Utrecht University, Utrecht, the Netherlands; 4 Viroclinics Biosciences B.V., Rotterdam, the Netherlands; University of Kansas Medical Center, United States of America

## Abstract

Using random PCR in combination with next-generation sequencing, a novel parvovirus was detected in the brain of a young harbor seal (*Phoca vitulina*) with chronic non-suppurative meningo-encephalitis that was rehabilitated at the Seal Rehabilitation and Research Centre (SRRC) in the Netherlands. In addition, two novel viruses belonging to the family *Anelloviridae* were detected in the lungs of this animal. Phylogenetic analysis of the coding sequence of the novel parvovirus, tentatively called Seal parvovirus, indicated that this virus belonged to the genus *Erythrovirus*, to which human parvovirus B19 also belongs. Although no other seals with similar signs were rehabilitated in SRRC in recent years, a prevalence study of tissues of seals from the same area collected in the period 2008-2012 indicated that the Seal parvovirus has circulated in the harbor seal population at least since 2008. The presence of the Seal parvovirus in the brain was confirmed by real-time PCR and *in vitro* replication. Using *in situ* hybridization, we showed for the first time that a parvovirus of the genus *Erythrovirus* was present in the Virchow-Robin space and in cerebral parenchyma adjacent to the meninges. These findings showed that a parvovirus of the genus *Erythrovirus* can be involved in central nervous system infection and inflammation, as has also been suspected but not proven for human parvovirus B19 infection.

## Introduction

Parvoviruses are small, non-enveloped viruses containing a single-stranded DNA genome of approximately 5 kb [1]. The family *Parvoviridae* includes the subfamily *Parvovirinae*, members of which infect vertebrates, and the subfamily *Densovirinae*, members of which infect invertebrates. Five genera of the subfamily *Parvovirinae* have been recognized by the International Committee on the Taxonomy of Viruses (ICTV), *Amdovirus*, *Bocavirus, Dependovirus*, *Erythrovirus* and *Parvovirus* [2]. In addition, two new genera (*Bufavirus* and *Partetravirus*) have been proposed within this subfamily [3,4]. Dependent on host, immune status, and parvovirus species, clinical manifestations of parvovirus infection may range from subclinical infection to severe diseases, including abortion, chronic immune complex disease, enteritis, hepatitis and myocarditis [1]. 

The genus *Erythrovirus* consists of four species (Human parvovirus B19 and those which have so far only been identified in primates: Pig-tailed macaque parvovirus, Rhesus macaque parvovirus and Simian parvovirus) and two tentative members (Bovine parvovirus type 3 and Chipmunk parvovirus) [2]. Human parvovirus B19 is the best studied member of this genus. This virus was identified as the cause of fifth disease, a rash that occurs mainly in young children (for review see [5,6]:). In addition, human parvovirus B19 has been associated with severe hematological disorders, hydrops fetalis upon infection during pregnancy and it was detected in cerebrospinal fluid (CSF) and serum from patients with meningo-encephalitis [5,7–9]. However, the exact pathogenesis of human parvovirus B19 has not been fully elucidated [5]. In macaques, erythroviruses were identified in animals with anemia [10,11], while Bovine parvovirus type 3 and Chipmunk parvovirus were identified in apparently healthy animals [12,13]. Upon experimental inoculation of cynomolgus macaques with Simian parvovirus, macaques developed mild clinical signs including transient cessation of erythropoiesis confirming the role of this virus in the observed anemia [14]. 

In the present study, we described the identification, partial characterization, and prevalence of a newly discovered parvovirus, tentatively classified within the genus *Erythrovirus* and called Seal parvovirus, that we detected in various organs, including the brain, of a harbor seal with chronic brain disease.

## Results

### Macroscopic and microscopic observations

**Figure 1:**
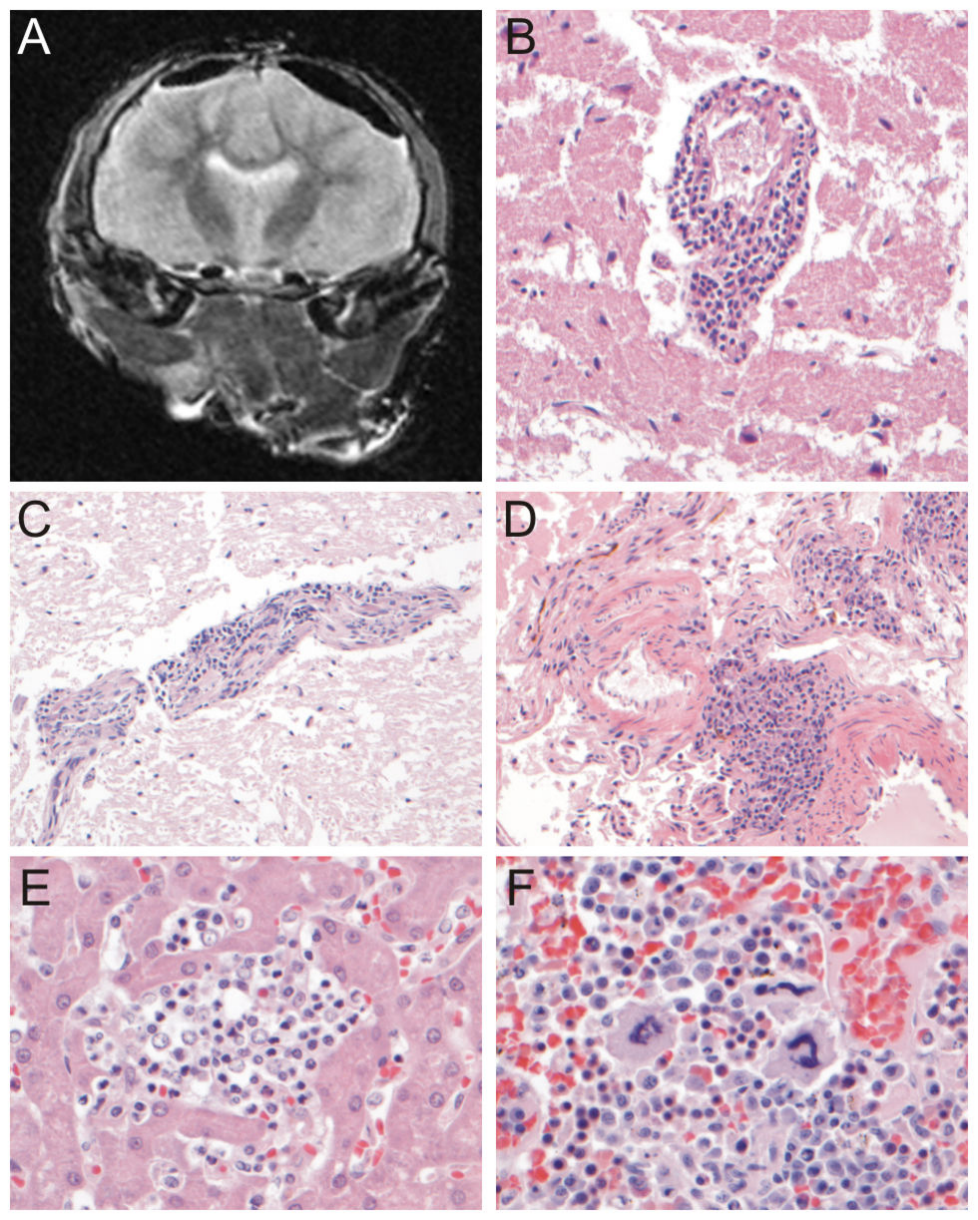
Evaluation of the presence of lesions by MRI and histology. A.Transverse T2-weighted MR image of the brain of seal 12-410. No gross abnormalities were detected in the brain parenchyma (the subdural hypointense areas are compatible with air due to post-mortem preparation). B, C, D. Perivascular cuffing with inflammatory cells distending the Virchow-Robin space in the brain parenchyma (B, C) and in the meninges (D). The infiltrates consisted mainly of mononuclear cells. E. Infiltrates of neutrophils and mononuclear cells in the liver parenchyma. F. Megakaryocytes and rubricytes in the spleen associated with extramedullary hematopoiesis. H&E stained slides, original magnification B, C, D: 200x; E, F: 400x.

External examination of seal 12-410 at necropsy revealed numerous ulcerated raised skin lesions varying in size from 5mm to several centimetres in diameter, mainly located at the ventral side of the body and on the right front flipper. Internal examination showed multiple firm dark red foci in the lungs. No further gross abnormalities were observed in the brain or other organs (Figure **1A**). On MRI images of the brain, subdural areas with a hypointense signal were found on all sequence images, compatible with gas formation due to decomposition. Upon histological examination, multifocally in the cerebrum, cerebellum, brainstem, choroid plexus and meninges a mild chronic non-suppurative meningo-encephalitis was observed, characterized by the presence of a mononuclear perivascular infiltrate consisting predominantly of lymphocytes up to 5 cell layers thick (Figure **1B, C, D**). Inflammatory cells did not extend into the brain parenchyma surrounding affected blood vessels. The skin had multifocal chronic severe proliferative and necrotizing dermatitis with eosinophilic intracytoplasmic inclusion bodies predominantly in acanthocytes, which might have been caused by infection with Seal poxvirus [15]. The lungs showed multifocal to coalescing chronic moderate pyogranulomatous and eosinophilic bronchopneumonia with intralesional nematodes. Such parasitic bronchopneumonia was commonly observed in young harbour seals in this population [16,17]. In the liver, mild hepatitis was observed characterized by the presence of multifocal aggregates of small numbers of neutrophils and mononuclear cells in portal areas and in distended sinusoids, infrequently associated with hepatocyte necrosis (Figure **1E**). Multifocally in the spleen and to a lesser extent in the liver, extramedullary haematopoiesis was present, characterized by the presence of precursors of the eyrthroid series and megakaryocytes (Figure **1F**). No histological abnormalities were detected in remaining organs. 

### Discovery of a novel parvovirus and two novel anelloviruses

**Figure 2:**
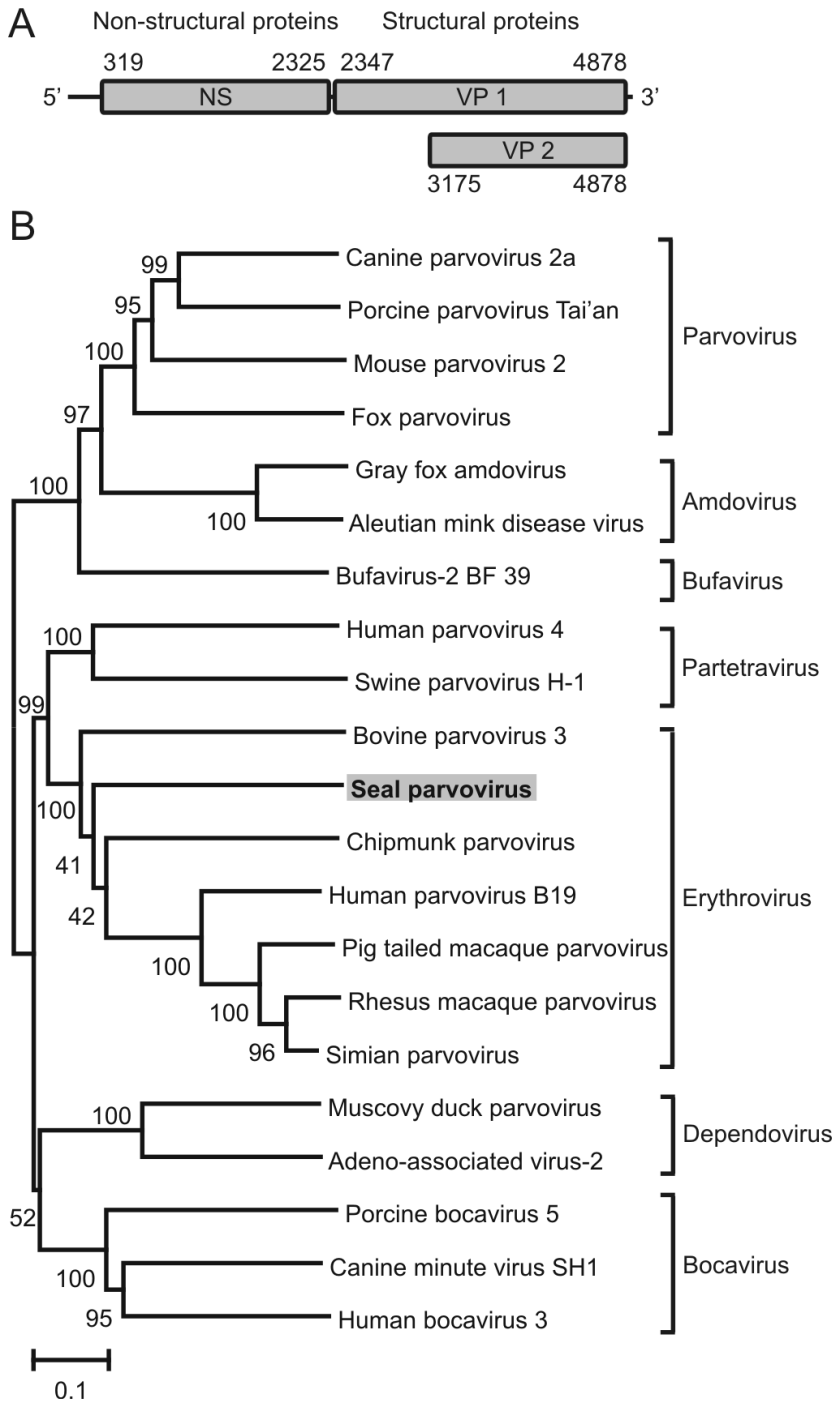
Genome organization and phylogenetic analysis of Seal parvovirus. A. Genome organization of Seal parvovirus. Indicated were the location of the major ORFs (grey) and the location of the start- and stopcodons on the nucleotide level counted from the 5’ end of the partial seal parvovirus genome. B. Phylogenetic neighbor-joining tree with *p*-distance and 1,000 bootstrap replicates of the deduced amino acid sequences of the VP2 genes of various viruses of the subfamily *Parvovirinae*. Genbank accession numbers: Canine parvovirus 2a: JQ996152, Porcine parvovirus Tai’an: FJ853421, Mouse parvovirus 2: NC_008186, Fox parvovirus: KC692368, AMD (Aleutian Mink disease) parvovirus: GU183264, Gray fox amdovirus: JN202450, Bufavirus-2 BF 39: JX027297, Human parvovirus 4: AY622943, Swine parvovirus H-1: AB076669, Bovine parvovirus 3: AF406967. Seal parvovirus: KF373759, Chipmunk parvovirus: GQ200736, Pig tailed macaque parvovirus: AF221123, Rhesus macaque parvovirus: AF221122, Simian parvovirus: U26342, Human parvovirus B19: NC_000883, Muscovy duck parvovirus: NC_006147, Adeno-associated virus-2: NC_001401, Porcine bocavirus 5: JN831651, Canine minute virus SH1: FJ899734, Human bocavirus 3: HM132056.

No herpesvirus and morbillivirus were detected by specific PCR and isolated nucleic acids were further processed for random PCR in combination with next-generation sequencing. More than 70,000 reads were analyzed from the lungs and brain of seal 12-410. Using BLAST analysis comparing these sequences, 81 sequences were detected that showed the closest similarity with Chipmunk parvovirus in the lung and brain and 20 sequences were detected that showed the closest similarity with Seal anellovirus TFFN/USA/2006 in the lung [12,18]. The coding sequence of a novel parvovirus, tentatively called Seal parvovirus, was obtained by Sanger sequencing (Genbank accession number KF373759) in which three major open reading frames were identified. The first ORF encoded the putative non-structural (NS) protein (nt 319 to 2325), the second ORF encoded the structural protein VP1 (nt 2347 to 4878), and the third ORF (nt 3175 to 4878), identified within the VP1 gene, coded for the structural protein VP2 (Figure **2A**). Phylogenetic analysis of the deduced complete amino acid sequence of the VP2 and NS1 protein revealed that this novel parvovirus most likely belonged to the genus *Erythrovirus* (Figure **2B** **+ Figure S1**). Analysis of the pairwise nucleotide and deduced amino acid sequence identities between the NS1 and VP2 gene of the novel Seal parvovirus and various other viruses of the subfamily *Parvovirinae* revealed that the VP2 gene had the highest identity with the Chipmunk parvovirus (only 39% on the deduced amino acid level), while the NS1 gene had the highest identity with the Pigtailed macaque parvovirus (only 27% on the deduced amino acid level) (Table **S1** and **S2**).

**Figure 3:**
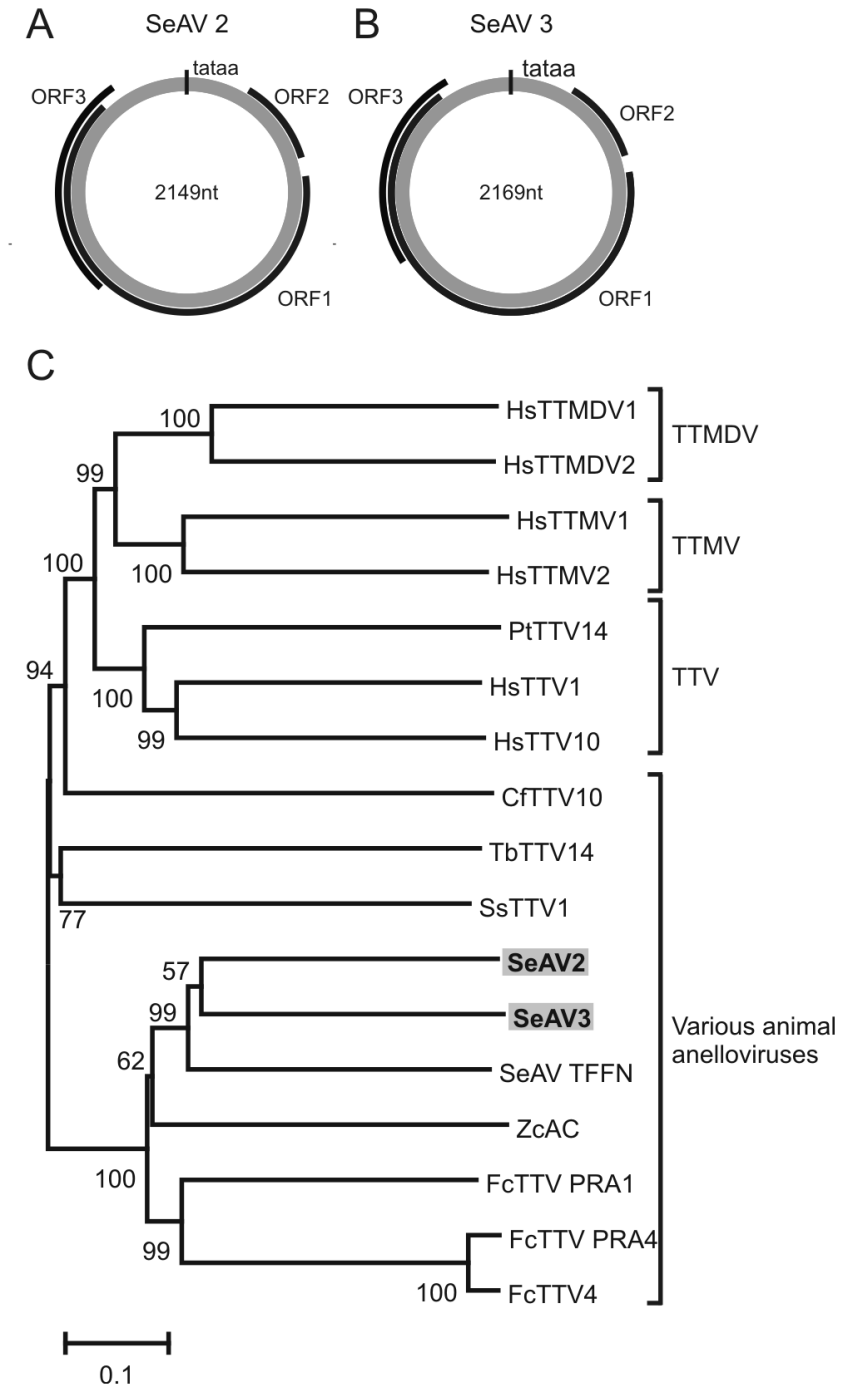
Genome organization and phylogenetic analysis of Seal anelloviruses 2 and 3. A, B Genome organization of the two novel anelloviruses, Seal anellovirus 2 (A) and Seal anellovirus 3 (B). The location of the three main ORFs was indicated (black line). C. Phylogenetic neighbor-joining tree with *p*-distance and 1,000 bootstrap replicates of the deduced amino acid sequences of the ORF1 genes of representative viruses of the family *Anelloviridae*. Genbank accession numbers: HsTTMDV1 (Homo sapiens torque teno midi virus 1): NC_009225, HsTTMDV2 (Homo sapiens torque teno midi virus 2): NC_014093, HsTTMV1 (Homo sapiens torque teno mini virus 1): NC_014097, HsTTMV2 (Homo sapiens torque teno mini virus 2): NC_014086, PtTTV14 (Pan troglodytes torque teno virus 14): NC_014077, HsTTV1 (Homo sapiens torque teno virus 1): NC_007013, HSTTV10 (Homo sapiens torque teno virus 10): GU797360, CfTTV10 (Canis familiaris torque teno virus 10): NC_014071, TbTTV14 (Tupaia belangeri chinensis torque teno virus 14): AB057358, SsTTV1 (Sus sucrofa torque teno virus 1): AY823990, SeAV2 (Seal anellovirus 2): KF373760 , SeAV3 (Seal anellovirus 3): KF373758, SeAv TFFN (Seal anellovirus TFFN/USA/2006): NC_015212, ZcAV (Zalophus californianus torque tenovirus): FJ459582, FcTTV4 (Felis catus torque teno virus 4): AB076003, PRA4 (Felis catus anellovirus PRA 4): EF538878, PRA1 (Felis catus anellovirus PRA1): EF538877.

In addition to the newly discovered Seal parvovirus, two novel anelloviruses were detected and the complete sequences of these viruses, tentatively called Seal anelloviruses 2 and 3 (Genbank accessions KF373760 and KF373758), were also obtained. Three major ORFs and a TATA-box were identified in both anelloviruses, with a similar genome organization as the anellovirus detected in seals previously [18] (Figure **3A, B**). A phylogenetic tree based on the deduced amino acid sequence of the ORF1 sequences of Seal anelloviruses 2 and 3 revealed that both viruses were closely related to Seal anellovirus TFFN/USA/2006 (unclassified anellovirus) displaying respectively only 58% and 52% identity on the nucleotide level in ORF1 (Figure **3C**). Genera in the family *Anelloviridae* are defined as having >56% divergence in the nucleotide sequence of ORF1 [19], indicating that Seal anelloviruses 2 and 3 belonged to the same anellovirus genus as Seal anellovirus TFFN/USA/2006.

### Seal parvovirus detection by real-time PCR, ISH and *in vitro* replication

**Figure 4:**
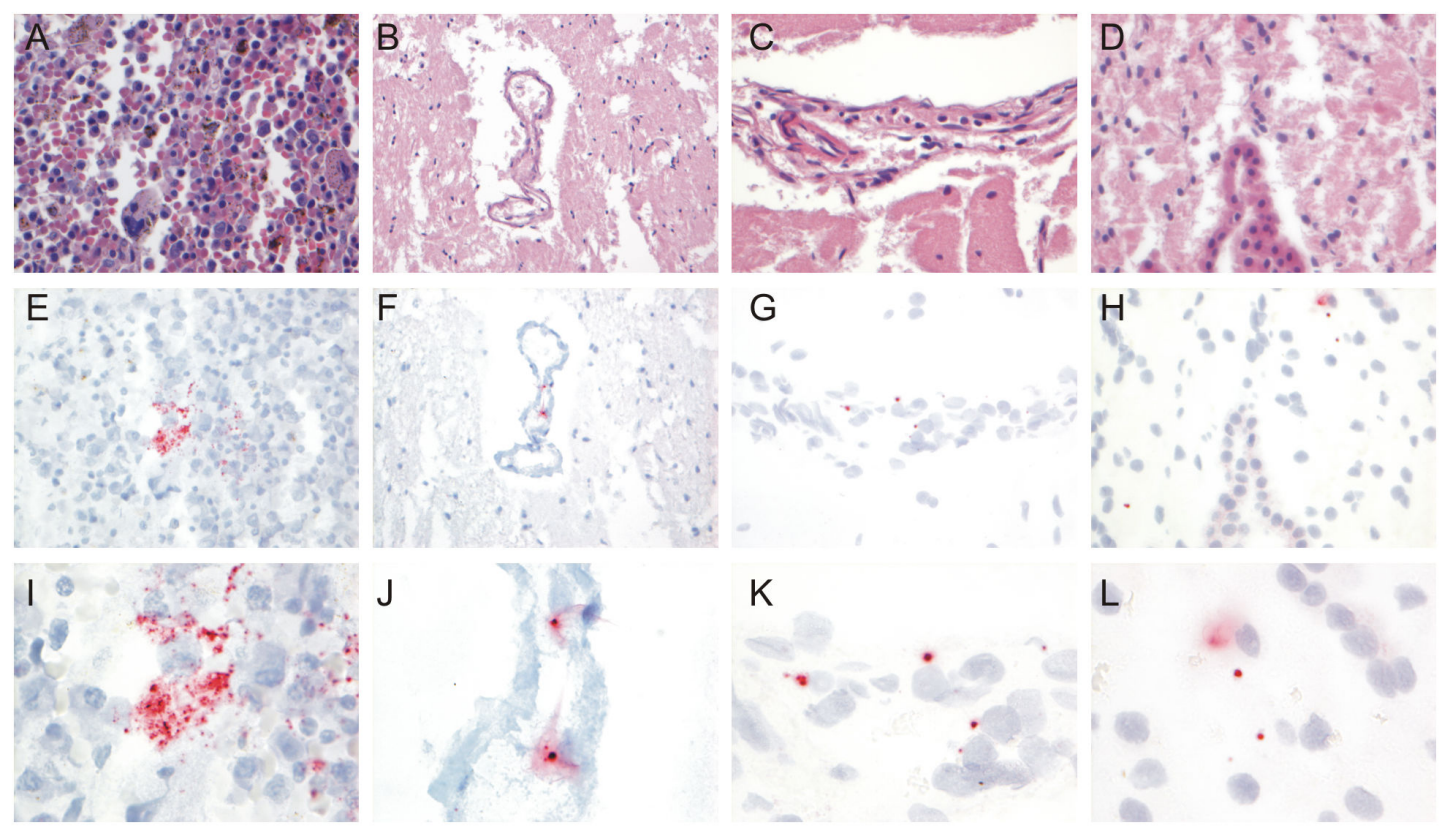
Detection of Seal parvovirus DNA by ISH. Pictures of H&E stained tissues of seal 12-410 of the spleen (A), the parenchyma of the cerebrum with a blood vessel (B), the Virchow-Robin space of the cerebellum (C) and the parenchyma of the cerebellum adjacent to the meninges (D). Red dots that indicate the presence of Seal parvovirus DNA were detected in the red pulp of the spleen (E, I), blood vessels in the brain parenchyma of the cerebrum (F, J), the Virchow-Robin space (G, K) and the brain parenchyma adjacent to the meninges of the cerebellum (H, L). A, B, C, D, E, F, G, K: original magnification 200x. I, J, K, L: original magnification 1000x.

In all tissues collected from seal 12-410, Seal parvovirus DNA was detected by real-time PCR with Ct-values ranging from 19 (spleen and lung) to 28 (brain stem) (Table **1**). In addition, Seal parvovirus DNA was detected in serum of this seal collected shortly after arrival at the SRRC and in serum collected at the moment of euthanasia (37 days after collection of the original sample) (Table **1**). No Seal parvovirus DNA was detected by real-time PCR in the brain of another seal without neurological signs, that died at about the same time. The presence of Seal parvovirus DNA in the spleen and cerebrum and cerebellum was confirmed by ISH. Multifocally, Seal parvovirus DNA-probe signal was detected in the red pulp of the spleen (Figure **4A, E, I**), in blood vessels in the brain parenchyma (Figure **4B, F, J**), in the Virchow-Robin spaces of the cerebrum (Figure **4C, G, K**) and in the brain parenchyma adjacent to the choroid plexus (Figure **4D, H, L**). No clear signal could be detected in the lungs and liver of seal 12-410, in tissues of the same seal incubated with the negative control probe and in tissues of the negative control seal (data not shown). 

**Table 1:** Summary of tissue distribution of Seal parvovirus DNA in three positive seals.

Year		2008	2012 (12-410)	2012
Clinical signs		Unknown	Hemiparesis, unconsiousness	Pneumonia
Tissues tested for Seal parvovirus DNA by real-time PCR*	Cerebrum	-	+ (24)	+ (31)
	Cerebellum	n.a.	+ (22)	n.a.
	Brain stem	n.a.	+ (28)	n.a.
	Lung	+ (26)	+ (21)	+ (37)
	Spleen	+ (35)	+ (19)	+ (22)
	Kidney	n.a.	+ (21)	n.a.
	Liver	n.a.	+ (23)	+ (37)
	Serum	n.a.	+ (27-32)#	n.a.
Detection of Phocine herpesvirus 1 DNA		+	-	+

* Ct-values are indicated in brackets

n.a.: not available

^#^ Range of Ct-values of Seal parvovirus DNA detected in serum that was collected at various timepoints from admission of the seal to the SRRC until euthanasia.

**Figure 5:**
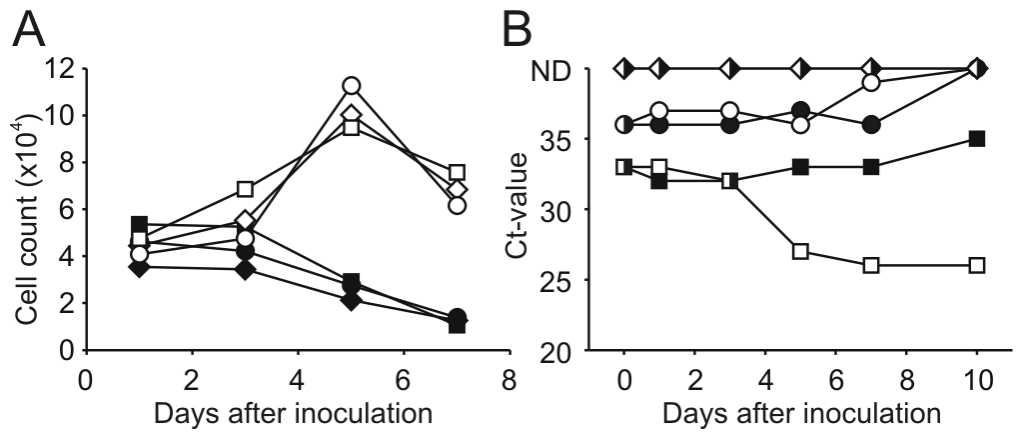
Replication of Seal parvovirus *in* *vitro*. Bone marrow cells of seal were incubated with (open symbols) or without (closed symbols) canine erythropoietin and inoculated with either PBS (diamond) or 2µl (circles) or 20µl (squares) of brain tissue homogenate from the parvovirus positive seal. At various time points after inoculations, samples were collected for counting of live cells by FACSanalysis (A) or real time-PCR using primers and probe specific for the Seal parvovirus (B). The detection limit of the assay was indicated with ND (not detectable).

The presence of infectious Seal parvovirus in the brain of seal 12-410 was confirmed by *in vitro* culture using seal bone marrow cells. The number of cultured cells increased and erythropoiesis was induced in the presence of cEPO, which was not the case in the absence of cEPO (Figure **5A**, data not shown). Following inoculation of bone marrow cells with lung tissue homogenate, cells died rapidly, most likely due to the presence of toxic components (data not shown). No increased cell death was observed when brain tissue homogenate was added (Figure **5A**). Ct-values of cells inoculated with 40μl/ml brain tissue and cEPO decreased from 33 at t=0 to 26 at t=7 and t=10, suggesting active virus replication (Figure **5B**). No Seal parvovirus DNA was detected in cells incubated with transport medium only, with the same homogenate without cEPO, or in cells incubated with 4μl/ml control brain tissue homogenate with cEPO. 

### Prevalence of Seal parvovirus in harbor seals of the Dutch coastal waters

To determine whether Seal parvovirus was present in the harbor seal population in the Dutch coastal waters in recent years, samples of spleens, brains and lungs of 94 additional harbor seals on which a necropsy was performed at the SRRC from 2008-2012 were collected and tested for the presence of Seal parvovirus DNA by real-time PCR (Table **2**). Seal parvovirus DNA was detected in several tissues of two harbor seals in addition to seal 12-410, one in 2008 and one in 2012 (Tables **1** and **2**). In tissues of both of these additional seals, Phocine herpesvirus 1 DNA was detected by PCR. In the seal that had died in 2012, Seal parvovirus DNA was also detected in the brain, although with a higher Ct-value than in seal 12-410 (Table **1**). 

**Table 2:** Summary of animals tested for Seal parvovirus DNA.

Year	2008	2009	2010	2011	2012	Total
Pup	0/3*	0/1	0/0	0/1	0/2	0/6
Juvenile	0/12	0/13	0/16	0/12	2/14	2/67
(Sub)adult	0/4	0/1	0/2	0/5	0/4	0/26
Unknown	1/1	0/0	0/2	0/2	0/0	1/5
Total number	1/20	0/15	0/20	0/20	2/20	3/95

* Number of Seal parvovirus DNA positive seals/ number of seals tested

## Discussion

Various outbreaks of disease in marine mammals have been the subject of virus discovery activities in the past decades. Although influenza A viruses, morbilliviruses and a herpesvirus were identified as major pathogens of harbor seals [20–22], information about the presence of other viruses and their pathogenicity is still limited. Recently, a novel anellovirus, picornavirus and herpesviruses were detected during outbreaks of disease [18,23,24]. Although parvoviruses are known to be a major cause of disease of several terrestrial carnivore species [25], they have not yet been detected in harbor seals. In the present study, we described the detection and partial characterization of a novel parvovirus, tentatively called Seal parvovirus, in the brain of a harbor seal with severe chronic neurological signs. In addition to this parvovirus, we also discovered two novel anelloviruses (Seal anellovirus 2 and 3) in the lungs of the same seal. Since anelloviruses are ubiquitous and their causative role in neurological disease was not considered likely [19,26], we primarily focused on the potential role of Seal parvovirus in the brain disease in the present case.

The observed progressive clinical signs in seal 12-410 indicated CNS disease, which was confirmed by the presence of a chronic meningo-encephalitis observed by histological examination. We excluded the presence of several other causes of brain disease by MRI (e.g. tumor or trauma) and by PCR for morbilliviruses and herpesviruses, both important infectious causes of encephalitis in harbor seals [27,28]. The non-suppurative character of the meningo-encephalitis did not fit with most bacterial causes. In addition, by random PCR in combination with deep sequencing no bacterial sequences known to be associated with meningo-encephalitis were detected. Seal parvovirus DNA was detected retrospectively in the serum of this animal already upon admission to the SRRC, confirming the presence of an infection with this virus concurrent with the observed left-sided hemiparesis upon admission. Since the presence of the newly identified Seal parvovirus was also detected in the brain of this animal by real-time PCR and in-situ hybridisation, it is likely that the parvovirus infection played a role in the development of the neurological disease of this animal. However, Koch’s postulates would have to be fulfilled to formally prove the role of this virus in the disease. 

Human parvovirus B19, a phylogenetically closely related virus, has been associated with meningo-encephalitis in humans, mainly with underlying disease [7,8]. In all these human cases Parvovirus B19 DNA infection of the brain was diagnosed by PCR in CSF or serum [7,29], indicating that this is the first report that indeed shows that parvoviruses of the genus *Erythrovirus* can be present in the Virchow-Robin space and in the brain parenchyma adjacent to the meninges in mammals. Since we used a probe targeting DNA and not RNA to be able to detect a signal and we did not detect Parovirus DNA in the nuclei of cells, additional studies using RNA probes are of interest to elucidate whether viral replication might occur in dividing cells of the brain. *In vitro* experiments suggested that infectious viral particles were present in the brain tissue of seal 12-410. The observed decrease of the Ct-value from 33 to 26 suggests an approximately 150-fold increase in the amount of viral DNA based on calibration experiments (data not shown), which is similar to previous *in vitro* infection experiments with human parvovirus B19 and human bone marrow cells [30]. However, we were not able to repeat this experiment with fresh bone marrow cells from another seal that died at the SRRC, indicating that the *in vitro* infection system is rather inefficient. In addition, no cytopathic effect was observed which might be related to the relatively low number of infected cells *in vitro*. The absence of visible cytopathic effect does not exclude that this virus is indeed pathogenic in vivo, since many viruses do not cause visible cytopathic effect but are still pathogenic [31,32]. In addition to the brain, Seal parvovirus DNA was detected in various other tissues, including the lung, the liver and the spleen. While Parvovirus DNA could be detected in the spleen by ISH, no strong positive signal was detected in the liver and lungs. The relatively long storage of these tissues in formalin might have impaired the signal. Of interest, extramedullary hematopoiesis was observed in the spleen of this seal, which could have been caused by the chronic infection with Seal parvovirus as was demonstrated for other members of the genus *erythrovirus* [6,10,11]. However, extramedullary haematopoiesis was also observed in young pinnipeds without clinical signs [33].

No other seals were admitted to the SRRC in the past few years with similar clinical signs, suggesting that this case is probably a relatively unique event possibly associated with the presence of unknown host factors, similar to parvovirus-B19-associated meningo-encephalitis in humans [7,8]. Parvovirus DNA was detected in two other seals, one in 2008 and one in 2012, suggesting that this virus must have circulated among harbor seals of the Dutch coastal waters at least as early as 2008. However, both seals were co-infected with Phocine herpesvirus-1. Of interest, co-infection of humans with various pathogens and parvovirus B19 has been associated with progression to more severe disease previously [34–36]. The role of Seal parvovirus in the disease of the two other animals remained uncertain and should be the focus of additional studies.

In conclusion, we have described a seal with chronic non-suppurative meningo-encephalitis associated with infection with a novel parvovirus, tentatively named Seal parvovirus. These findings suggest that parvoviruses of the genus *Erythrovirus* can be involved in CNS inflammation, as has been suggested for Human parvovirus B19 [7,8]. The exact pathogenesis of this viral infection and the possible role of various host factors and co-infection with other pathogens should be the focus of future research.

## Materials and Methods

### Ethics statement

All samples of seals used in the present study were provided by the SRRC, and the SRRC provided permission to the Department of Viroscience, Erasmus Medical Centre to use the samples for the present study. Admission and rehabilitation of wild seals at the SRRC and collection of wild dead seals for diagnostic purposes by the SRRC is permitted by the government of the Netherlands (application number FF/75/2012/015). In the present study, only samples were used that were collected from dead seals. These samples were collected from seals that were either found dead in the wild by the SRRC, died at the SRRC despite intensive care or were euthanized at the SRRC due to the presence of severe clinical signs in the absence of any indication of future recovery. In case of euthanasia, this was performed with T-61 (0.3ml/kg) after sedation as described previously [37]. No seals were euthanized for research purposes.

### Case description

A young male harbor seal (*Phoca vitulina*; approximately one year of age; rehabilitation number 12-410) stranded on the Dutch coast and was admitted to the Seal Rehabilitation and Research Centre (SRRC) in 2012. Upon arrival at the centre, the seal displayed left-sided hemiparesis. Despite treatment with antibiotics and anti-inflammatory medication for several weeks, the seal developed severe central nervous system (CNS) sings including episodes of unconsciousness. Since no recovery was observed, the seal was euthanized as described previously [37]. 

### Sample collection and evaluation of the presence of (histo)pathologic changes

Following euthanasia, the carcass was stored at -20°C for a few weeks. Subsequently, it was subjected to necropsy upon thawing and samples of brain, lungs, liver, spleen, kidney and urinary bladder were collected and stored at either -70°C or fixed in 10% neutral buffered formalin. In addition, the complete head was stored at -20°C for several weeks until further analysis by magnetic resonance imaging (MRI). A MRI study of the cranium was performed using a 0.2 Tesla open magnet (Magnetom Open Viva, Siemens AG, Munich, Germany). T1-weighted spin echo, T2 and proton density weighted turbo spin echo, Fluid Attenuated Inversion Recovery (FLAIR), Short Tau Inversion Recovery (STIR) and T1-weighted 3D gradient echo sequences were acquired in the transverse plane. Dual Echo Steady State (DESS) images were acquired in the dorsal plane. Subsequently, multiple samples of the brain stem, cerebellum and cerebrum were collected and stored at -70°C or fixed in 10% neutral-buffered formalin. After fixation, samples collected from various tissues were embedded in paraffin and 4- μm-thick tissue sections were stained with hematoxylin and eosin and evaluated for the presence of histological lesions.

### Processing of samples for specific PCR and random PCR in combination with next-generation sequencing

Tissues collected of seal 12-410 were tested for the presence of herpesvirus (lung, brain) and morbillivirus (brain) using a pan-herpes PCR and pan-morbillivirus PCR, respectively, as described previously [38,39]. In addition, samples of lungs and brain were processed for sequence independent RNA and DNA virus screening as described previously [40–42]. In brief, tissues were homogenized using a Fastprep-24 Tissue Homogenizer (MP Biomedicals) in Hank's balanced salt solution containing 0.5% lactalbumin, 10% glycerol, 200 U/ml penicillin, 200 μg/ml streptomycin, 100U/ml polymyxin B sulfate, 250 μg/ml gentamycin, and 50 U/ml nystatin (ICN Pharmaceuticals) (transport medium) and centrifuged briefly. Supernatants from tissue homogenates were filtered and treated with nucleases to decrease host DNA and RNA, after which RNA and DNA were extracted. After first and second strand synthesis, random amplification was performed and amplicons were processed for next-generation sequencing with a 454 GS Junior instrument (Roche). Reads were trimmed and assembled with *de novo* assembly using CLC Genomics Workbench 5 (CLC Bio), and analyzed by nucleotide and translated nucleotide BLAST searches. Sequences were classified based on the taxonomic origin of the best-hit sequence with MEGAN 4.70.4 [43], using E-value cut-offs of 0.001 and 10^-10^ for BLASTn and BLASTx searches, respectively. Obtained reads were deposited at the European Nucleotide Archive under archive number ERP003897.

### Genome sequencing and phylogenetic analysis

Using specific primers based on obtained 454-sequences, partially overlapping PCR amplicons spanning the near full-length genome of the novel seal parvovirus were obtained. To obtain 5’ and 3’ distal parts of the coding sequences of the major ORFs, parvovirus DNA was circularized using T4 DNA ligase and ligated 5’ and 3’ ends were amplified by specific primers. Amplicons were cloned into pCR4 Topo vector (Invitrogen) and sequences of at least two clones in two directions were obtained. Primer sequences are available upon request. The complete genome of two novel anelloviruses was obtained by rolling circle amplification using the Illustra Templiphi 100 amplification kit (GE Healthcare) with specific primers according to the recommendations of the manufacturer. PCR amplicons were separated by gel electrophoresis and bands of the right size were processed by sequence independent DNA amplification in combination with next-generation sequencing as described above. Obtained reads were analysed with the CLC Genomics Workbench 5 software and consensus sequences of the two novel anelloviruses were obtained. Phylogenetic analysis was performed by creation of multiple alignments using the ClustalW method in MEGA5 [44] based on the deduced amino acid sequences of the VP2 genes of the detected parvovirus and other representative parvoviruses and ORF1 of the novel anelloviruses and other anelloviruses [44]. Neighbour-joining phylogenetic trees were build with the *p*-distance model, 1000 bootstrap replicates, and otherwise default parameters in MEGA5 [44]. 

### Detection of Seal parvovirus in the tissue of seal 12-410 by real-time PCR and *in situ* hybridization

Using Primer Express software (version 3.0, Applied Biosystems) a real-time PCR was developed targeting a small fragment of the VP1 gene of the novel parvovirus, using forward primer 5’-CCTCGCAGGCATTTTCATG-3’, reverse primer 5’-TGCAAGAGCATCCACGGATA-3’ and probe 5’-FAM-AAACACAATCCTGACCGAGA-BHQ-3’. Amplification and detection of the Seal parvovirus DNA in nucleic acids of collected tissues was performed using a 7500 Real Time PCR system (Applied Biosystems) and Taqman Universal Mastermix (Applied Biosystems). All real-time PCRs were performed *in duplo*.

In addition to the real-time PCR, tissue sections of the cerebrum, cerebellum, lungs, spleen and liver of seal 12-410 were examined for the presence of the Seal parvovirus DNA by *in situ* hybridization (ISH) using a specific probe targeting the VP1 gene of Seal parvovirus and the RNAscope 2.0 kit (Advanced Cell Diagnostics) essentially according to the instructions of the manufacturer. In brief, 5μm-thick tissue sections were deparaffinized, treated with alkylphenol ethoxylate (APE), boiled for 10 minutes and pre-treated with buffers provided by the manufacturer. Subsequently, the specific probe was hybridized for two hours at 40°C and the signal was amplified by six amplification steps and visualised with FastRed. Sections were counterstained with hematoxylin. In addition, formalin pigment in tissue sections of lungs and spleen was removed by pre-treatment of sections with APE. The quality of the tissues was confirmed using the Ubiquitin C control and slides processed with a DapB probe provided by the manufacturer. In addition, brain tissue sections of an age-matched control seal without neurological signs and undetectable Seal parvovirus DNA by real-time PCR were used as controls for the presence of a Seal parvovirus specific signal.

### Replication of newly discovered Seal parvovirus *in vitro*

The presence of infectious virus in brain and lung tissue of seal 12-410 was evaluated by inoculation of suspension cultures of fresh harbor seal bone marrow cells with brain and lung tissue homogenate essentially as described previously for human parvovirus B19 [30]. In brief, femur bones of a seal that had died at the SRRC from an apparently non-infectious cause, were collected, cleaned by storage in 70% ethanol for 5 minutes and rinsed twice with phosphate buffered saline (PBS). Subsequently, bones were stored in cold RPMI 1640 medium (Cambrex, East Rutherford, NJ) supplemented with 20% fetal bovine serum, 500IU/ml penicillin and 500μg/ml streptomycin (Culture medium) for three hours until bone marrow cells were collected by flushing out bone marrow with culture medium. Obtained cells were centrifuged, resuspended in PBS, and mononuclear cells were isolated by density gradient centrifugation using Lymphoprep solution (Axis-Shield, PoC AS, Oslo, Norway). Isolated cells were washed with PBS supplemented with 2.5mM EDTA, resuspended in RPMI 1640 medium supplemented with 500IU/ml penicillin and 500μg/ml streptomycin, and live cells were counted by trypan blue exclusion. 

Subsequently, 10x10^6^ cells/ml were inoculated with either 4 or 40 μl brain or lung tissue homogenate or transport medium (negative control). After storage at 4°C for 2 hours, culture medium with or without 40ng/ml recombinant canine erythropoietin (cEPO; R&D Systems, Minneapolis, USA) was added to a final concentration of 2x10^6^ cells/ml and cells were stored at 37ºC/5% CO_2_. At 0, 1, 3, 5, 7 and 10 days post inoculation, samples of the suspension cultures were collected for determination of the quantity of Seal parvovirus DNA using real-time PCR and at 1, 3, 5 and 7 days post inoculation for determination of the number of cells. Cell numbers were determined by acquiring a fixed volume of cells with a FACSCantoII (BD, Alphen a/d Rijn, the Netherlands) and analysed using FACS Diva Software (BD). 

### Prevalence study

Samples of spleens, brains and lungs of 94 additional harbor seals, on which a necropsy was performed at the SRRC from 2008-2012 were collected and tested for the presence of Seal parvovirus DNA by real-time PCR (Table **2**). Tested samples were collected from wild (n=16) and rehabilitated (n=71) harbor seals or were of unknown origin (n=7) and seals were divided into age groups as described previously [45]. No other seals were rehabilitated in the past few years with signs similar to those of seal 12-410, and severe (parasitic) bronchopneumonia was the main suspected cause of death (68%) of the seals that were screened for the presence of Seal parvovirus DNA.

## Supporting Information

Figure S1 **Phylogenetic analysis of the NS1 protein of Seal parvovirus.** Phylogenetic neighbor-joining tree with *p*-distance and 1,000 bootstrap replicates of the deduced amino acid sequences of NS1 genes of various viruses of the subfamiliy *Parvovirinae*. Significant bootstrap values are shown. Genbank accessions: Canine parvovirus CPV-N: M19296, Mouse parvovirus UT: AB234204, Porcine parvovirus Tai’an: FJ853421, Fox parvovirus: KC692368, Bufavirus-2 BF 39: JX027297, Gray fox amdovirus: JN202450, AMD (Aleutian Mink disease) parvovirus: GU183264, Human parvovirus 4: AY622943, Swine parvovirus H-1: AB076669, Adeno-associated virus-2: NC_001401, Duck parvovirus: NC_006147, Seal parvovirus: KF373759, Bovine parvovirus 3: AF406967, Chipmunk parvovirus: GQ200736, Human parvovirus B19: NC_000883, Pig tailed macaque parvovirus: AF221123, Simian parvovirus: U26342, Rhesus macaque parvovirus: AF221122, Porcine bocavirus 5: JN831651, Canine minute virus SH1: FJ899734, Human bocavirus 3: HM132056.
(TIF)

Table S1 **Nucleotide (NT) and deduced amino acid (AA) sequence identities (%) between the VP2 gene of Seal parvovirus and selected other parvoviruses of the genera *Erythrovirus, Partetravirus, Adeno-associated virus* and *Parvovirus*.**
(DOC)

Table S2 **Nucleotide (NT) and deduced amino acid (AA) sequence identities (%) between the NS1 gene of Seal parvovirus and selected other parvoviruses of the genera *Erythrovirus, Partetravirus, Adeno-associated virus* and *Parvovirus*.**
(DOC)
