# Supplementary figures and images for: Novel B19-Like Parvovirus in the Brain of a Harbor Seal
Source: PLoS One. 2013 Nov 5;8(11):e79259. doi: 10.1371/journal.pone.0079259 (PMC3818428; doi:10.1371/journal.pone.0079259)

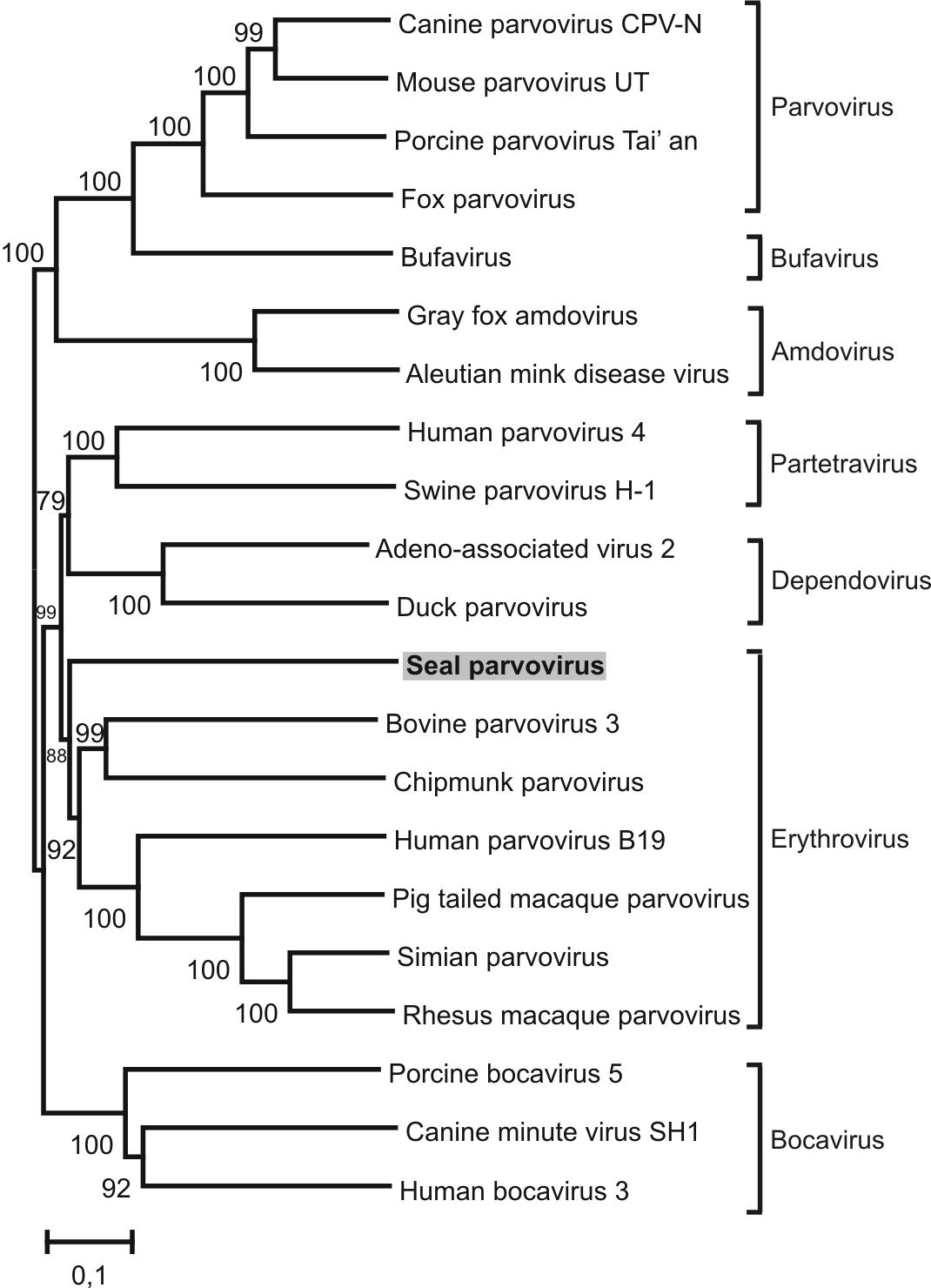

Supplement: Figure S1 — Phylogenetic analysis of the NS1 protein of Seal parvovirus. Phylogenetic neighbor-joining tree with p-distance and 1,000 bootstrap replicates of the deduced amino acid sequences of NS1 genes of various viruses of the subfamiliy Parvovirinae. Significant bootstrap values are shown. Genbank accessions: Canine parvovirus CPV-N: M19296, Mouse parvovirus UT: AB234204, Porcine parvovirus Tai’an: FJ853421, Fox parvovirus: KC692368, Bufavirus-2 BF 39: JX027297, Gray fox amdovirus: JN202450, AMD (Aleutian Mink disease) parvovirus: GU183264, Human parvovirus 4: AY622943, Swine parvovirus H-1: AB076669, Adeno-associated virus-2: NC_001401, Duck parvovirus: NC_006147, Seal parvovirus: KF373759, Bovine parvovirus 3: AF406967, Chipmunk parvovirus: GQ200736, Human parvovirus B19: NC_000883, Pig tailed macaque parvovirus: AF221123, Simian parvovirus: U26342, Rhesus macaque parvovirus: AF221122, Porcine bocavirus 5: JN831651, Canine minute virus SH1: FJ899734, Human bocavirus 3: HM132056. (TIF) [file pone.0079259.s001.tif]
