# Supplementary material for: Novel B19-Like Parvovirus in the Brain of a Harbor Seal
Source: PLoS One. 2013 Nov 5;8(11):e79259. doi: 10.1371/journal.pone.0079259 (PMC3818428; doi:10.1371/journal.pone.0079259)
Supplement: Table S1 — Nucleotide (NT) and deduced amino acid (AA) sequence identities (%) between the VP2 gene of Seal parvovirus and selected other parvoviruses of the genera Erythrovirus, Partetravirus, Adeno-associated virus and Parvovirus. (DOC) [file pone.0079259.s002.doc]

|  | **Seal parvovirus** | | Chipmunk parvovirus | | Human parvovirus B19 | | Muscovy duck parvovirus | | Adeno-associated virus 2 | | Canine parvovirus 2a | |
| --- | --- | --- | --- | --- | --- | --- | --- | --- | --- | --- | --- | --- |
|  | NT | AA | NT | AA | NT | AA | NT | AA | NT | AA | NT | AA |
| **Seal parvovirus** | - | - | 47 | 39 | 46 | 37 | 38 | 27 | 41 | 27 | 32 | 17 |
| Chipmunk parvovirus | 47 | 39 | - | - | 50 | 45 | 39 | 29 | 40 | 26 | 34 | 18 |
| Human parvovirus B19 | 46 | 37 | 50 | 45 | - | - | 41 | 32 | 39 | 26 | 33 | 16 |
| Pig-tailed macaque parvovirus | 45 | 36 | 49 | 41 | 67 | 68 | 39 | 30 | 37 | 25 | 34 | 16 |
| Rhesus macaque parvovirus | 42 | 34 | 47 | 39 | 64 | 67 | 39 | 29 | 38 | 24 | 34 | 15 |
| Simian parvovirus | 44 | 37 | 50 | 43 | 65 | 68 | 40 | 33 | 38 | 25 | 33 | 16 |
| Bovine parvovirus 3 | 42 | 31 | 45 | 36 | 50 | 43 | 36 | 25 | 39 | 25 | 34 | 17 |
| Human parvovirus 4 | 38 | 27 | 42 | 26 | 45 | 29 | 45 | 40 | 39 | 23 | 37 | 16 |
| Swine parvovirus H-1 | 38 | 27 | 39 | 29 | 41 | 32 | 38 | 24 | 39 | 24 | 30 | 16 |
| Muscovy duck parvovirus | 40 | 25 | 40 | 26 | 39 | 26 | - | - | 59 | 58 | 36 | 19 |
| Adeno-associated virus 2 | 41 | 27 | 40 | 26 | 39 | 26 | 59 | 58 | - | - | 32 | 19 |
| Canine parvovirus 2a | 32 | 17 | 34 | 18 | 33 | 16 | 36 | 19 | 32 | 19 | - | - |
