# Supplementary material for: Novel B19-Like Parvovirus in the Brain of a Harbor Seal
Source: PLoS One. 2013 Nov 5;8(11):e79259. doi: 10.1371/journal.pone.0079259 (PMC3818428; doi:10.1371/journal.pone.0079259)
Supplement: Table S2 — Nucleotide (NT) and deduced amino acid (AA) sequence identities (%) between the NS1 gene of Seal parvovirus and selected other parvoviruses of the genera Erythrovirus, Partetravirus, Adeno-associated virus and Parvovirus. (DOC) [file pone.0079259.s003.doc]

|  | **Seal parvovirus** | | Chipmunk parvovirus | | Human parvovirus B19 | | Pig-tailed macaque parvovirus | | Duck parvovirus | | Adeno-associated virus 2 | | Canine parvovirus CPV-N | |
| --- | --- | --- | --- | --- | --- | --- | --- | --- | --- | --- | --- | --- | --- | --- |
|  | NT | AA | NT | AA | NT | AA | NT | AA | NT | AA | NT | AA | NT | AA |
| **Seal parvovirus** | - | - | 39 | 25 | 36 | 23 | 39 | 27 | 36 | 21 | 40 | 26 | 32 | 16 |
| Chipmunk parvovirus | 39 | 25 | - | - | 41 | 29 | 39 | 26 | 39 | 24 | 41 | 29 | 35 | 17 |
| Human parvovirus B19 | 36 | 23 | 41 | 29 | - | - | 51 | 42 | 38 | 22 | 38 | 25 | 35 | 15 |
| Pig-tailed macaque parvovirus | 39 | 27 | 39 | 26 | 51 | 42 | - | - | 39 | 24 | 40 | 28 | 36 | 17 |
| Rhesus macaque parvovirus | 37 | 25 | 38 | 24 | 50 | 41 | 67 | 65 | 39 | 23 | 39 | 26 | 35 | 17 |
| Simian parvovirus | 37 | 24 | 39 | 25 | 52 | 43 | 67 | 67 | 42 | 25 | 40 | 27 | 35 | 16 |
| Bovine parvovirus 3 | 37 | 25 | 44 | 36 | 41 | 31 | 41 | 30 | 39 | 25 | 40 | 29 | 35 | 17 |
| Swine parvovirus H-1 | 37 | 21 | 36 | 22 | 35 | 24 | 36 | 23 | 38 | 25 | 41 | 26 | 30 | 15 |
| Human parvovirus 4 | 34 | 21 | 38 | 23 | 35 | 21 | 36 | 24 | 40 | 25 | 38 | 26 | 35 | 14 |
| Duck parvovirus | 36 | 21 | 39 | 24 | 38 | 22 | 39 | 24 | - | - | 54 | 50 | 36 | 19 |
| Adeno-associated virus 2 | 40 | 26 | 41 | 29 | 38 | 25 | 40 | 28 | 54 | 50 | - | - | 35 | 22 |
| Canine parvovirus CPV-N | 32 | 16 | 35 | 17 | 35 | 15 | 36 | 17 | 36 | 19 | 35 | 22 | - | - |
